# Periconceptional antibiotic use and early- to mid-pregnancy psychological distress in a nationwide birth cohort: cross-sectional analysis from the Japan Environment and Children’s Study

**DOI:** 10.1186/s12889-025-26119-0

**Published:** 2026-01-10

**Authors:** Kenta Matsumura, Hitomi Inano, Junko Sakai, Kanako Shimada, Akiko Tsuchida, Hidekuni Inadera, the Japan Environment and Children’s Study (JECS) Group, Michihiro Kamijima, Shin Yamazaki, Maki Fukami, Reiko Kishi, Koichi Hashimoto, Kenichi Sakurai, Shuichi Ito, Ryoji Shinohara, Takeo Nakayama, Ryo Kawasaki, Yasuhiro Takeshima, Hideki Nagashima, Mayumi Tsuji, Kimitoshi Nakamura

**Affiliations:** 1 Department of Epidemiology and Nutritional Psychiatry, Graduate School of Health Sciences, Aomori University of Health and Welfare, 58-1 Aza-Maze, Oaza Hamadate, Aomori City, 030-8505 Japan; 2 Department of Public Health, Faculty of Medicine, University of Toyama, Toyama City, Japan; 3 Toyama Regional Center for JECS, University of Toyama, Toyama City, Japan; 4 Department of Nursing Sciences, Graduate School of Medicine and Pharmaceutical Sciences, University of Toyama, Toyama City, Japan

**Keywords:** Antibiotic agent, Antibacterial drug, Psychological distress, Expectant mothers

## Abstract

**Background:**

Antibiotic use has recently emerged as a potential risk factor for psychiatric disorders, but evidence regarding its risk during pregnancy remains limited, despite the frequent use of antibiotics in pregnant women. We investigated the association between antibiotic use in the year prior to early pregnancy and psychological distress during early- to mid-pregnancy.

**Methods:**

Participants were 94,490 expectant mothers from the Japan Environment and Children’s Study, an ongoing nationwide birth cohort study. Antibiotic use during the year before early pregnancy was categorized as follows: (1) no use, (2) use during either the period before or after pregnancy recognition, and (3) use during both periods. Psychological distress was measured during early- to mid-pregnancy using the Kessler Psychological Distress Scale, with scores of 5–12 and ≥ 13 indicating moderate and severe psychological distress, respectively. A Bayesian multinomial generalized linear model was used to calculate adjusted odds ratios (aORs) and 95% credible intervals (95% CrIs), controlling for a priori selected potential confounders.

**Results:**

Analysis using no antibiotic use as a reference revealed that the aORs (95% CrIs) for moderate psychological distress were 1.07 (0.97–1.19) for use during either period and 1.22 (1.08–1.38) for use during both periods. For severe psychological distress, the aORs (95% CrIs) were 1.07 (0.97–1.19) and 1.50 (1.15–1.94), respectively.

**Conclusions:**

A dose–response-like pattern was observed, suggesting that even limited antibiotic use may be an independent risk factor for psychological distress during early- to mid-pregnancy, highlighting the importance of judicious antibiotic use from the preconception period.

**Supplementary Information:**

The online version contains supplementary material available at 10.1186/s12889-025-26119-0.

## Background

Perinatal depression is one of the most common psychiatric disorders that occur during pregnancy and after childbirth [1]. Its prevalence in high-income countries is approximately 10%–15% [2–4], and it is known to not only impair the mother’s quality of life but also negatively impact children’s development and family relationships [1, 2, 5, 6]. Additionally, maternal suicide, one of the leading causes of maternal mortality, is frequently associated with perinatal depression as a major comorbid condition [7, 8]. Reviews, including meta-analyses on postpartum depression, have consistently identified poor mental health during pregnancy as a strong risk factor [2, 9–12], making it a critical public health issue to prevent the deterioration of mental health during pregnancy.

The onset of perinatal depression is influenced by various factors, including psychopathological, socioeconomic, and environmental factors [10, 12, 13]. Among these, antibiotics, which are frequently used during the perinatal period [14], have recently emerged as a new risk factor for psychiatric disorders. A recent review article suggested that antibiotic use may worsen subsequent mental health outcomes [15]. For example, a large-scale UK-based study of more than 200,000 patients aged 15–65 years with depression found that an increase in the number of antibiotic courses was associated with a higher risk of depression and anxiety disorders [16]. Similar findings apply to pregnant and postpartum women. A prospective cohort study of mothers immediately after childbirth found that the use of antibiotics within 2 weeks postpartum was associated with an increased risk of depression at 1 and 2 months postpartum [17]. Additionally, another longitudinal study of women during pregnancy found that those with clinical depression had a higher rate of antibiotic use compared with those without [18]. Furthermore, our collaborator group has recently demonstrated that antibiotic use during pregnancy may increase the risk of postpartum depression, mediated by psychological distress during pregnancy [19]. A possible mechanism for these findings is the disruption of the gut microbiota by antibiotics [20]. This disruption has been associated with a variety of diseases, such as obesity, diabetes, and inflammation, and might also increase the risk of psychiatric disorders, such as depression and anxiety [21]. However, from a preventive perspective for postpartum depression, epidemiological evidence linking antibiotic use and maternal mental health remains limited. This is particularly true in terms of determining whether this relationship applies to preconception to early pregnancy use and its association with maternal mental health during pregnancy.

Therefore, in this study, we used data from a large-scale cohort study to investigate the association between antibiotic use during the past year prior to early pregnancy and psychological distress during early- to mid-pregnancy.

## Methods

### Study design and population

Participants were expectant mothers enrolled in the Japan Environment and Children’s Study (JECS), an ongoing nationwide prospective government-funded birth cohort study examining the impact of various environmental factors on children’s health and development. The design and baseline characteristics of the JECS have been reported in detail elsewhere [22, 23]. Briefly, pregnant women were enrolled between January 2011 and March 2014 via face-to-face recruitment at cooperating health care providers or Mother and Child Health Handbook delivery counters in 15 regional centers across Japan, covering both rural and urban areas. Their weeks of gestation at that point were a median [interquartile range (IQR)] of 12.3 [10.0, 15.6]. Participation was voluntary, and participants could withdraw at any time. Incentives (gift certificates) were provided for their participation. Follow-up was conducted during early- to mid-pregnancy (median [IQR]: 15.4 [12.4, 19.4] weeks of gestation). Data were collected from medical record transcriptions by physicians, midwives/nurses, and/or research coordinators and via self-report questionnaires distributed to the participants, as well as interviewer-administered questionnaires.

**Fig. 1:**
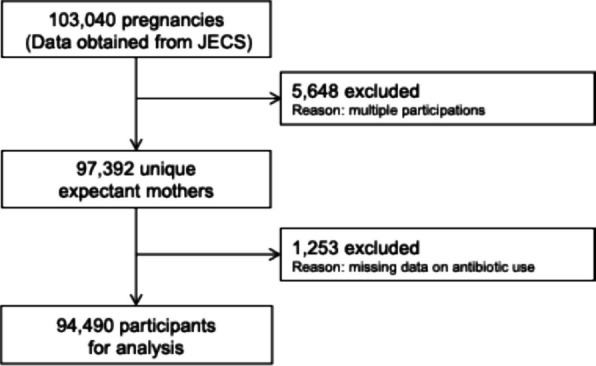
Participant flow diagram

The present study was based on data obtained from the JECS, which contains data on 103,040 pregnancies. In this study, 5,648 pregnancies were excluded due to multiple registrations (i.e., second or third registration of the same mother). Of the remaining 97,392 unique expectant mothers, an additional 2,902 were excluded due to missing data or lack of response regarding antibiotic use. This left 94,490 expectant mothers for the final analysis (Fig. 1).

All procedures contributing to this work complied with the ethical standards of the relevant national and institutional committees on human experimentation and with the 1975 Declaration of Helsinki, as revised in 2008. The JECS protocol was reviewed and approved by the Ministry of the Environment’s Institutional Review Board on Epidemiological Studies (100910001) and the ethics committees of all participating institutions. Written informed consent was obtained from all participants. The present study was also approved by the Ethics Committee of the University of Toyama (R2019035).

### Measures

#### Exposure

Periconceptional antibiotic use was assessed retrospectively through interviewer-administered questionnaires at enrollment (i.e., median [IQR]: 12.3 [10.0, 15.6] weeks of gestation, based on obstetric estimate) and was defined as any antibiotic agent administered during the past year. This 1-year interval was divided into two distinct periods: (1) from preconception to pregnancy recognition, and (2) from pregnancy recognition to enrollment. Mothers verbally reported all medications that they had taken, and research coordinators recorded and coded the responses using a drug dictionary.

Based on the collected data, antibiotic use was classified into three patterns: (1) no use, (2) use during either period, and (3) use during both periods.

#### Outcomes

Mental health status was measured using the Kessler Psychological Distress Scale (K6) [24] during early- to mid-pregnancy (i.e., median [IQR]: 15.4 [12.4, 19.4] weeks of gestation, based on obstetric estimate). The K6 is a 6-item self-report questionnaire developed to detect general psychological distress, written in a Likert-type format. Briefly, K6 items consist of feeling nervous, hopeless, fidgety, so depressed that nothing could cheer you up, that everything is an effort, and worthless. Participants were asked to mark their level of agreement with the response made on a 5-point response scale, with total scores ranging from 0 to 24. The Japanese version of the K6 was developed by Furukawa et al. [25], using a back-translation technique. It provides good screening performance equivalent to that of the K10 for mood and anxiety disorder diagnoses in the Diagnostic and Statistical Manual of Mental Disorders – Fourth Edition (DSM-IV) (area under the curve for both is 0.94) [25] and good internal reliability (Cronbach’s α = 0.84 for the current data). Its validity has been established with two cutoff points [25, 26]: 4/5, which is psychometrically the most appropriate (100% sensitivity and 69% specificity), and 12/13, which is the most widely used internationally (65% sensitivity and 97% specificity). Accordingly, we adopted a K6 score of 5–12 as the case definition of moderate psychological distress and a score of ≥ 13 as severe psychological distress [27, 28].

#### Potential confounders

We defined potential confounders as variables likely to affect antibiotic use and/or psychological distress, including basic demographic and socioeconomic characteristics, referring to previous studies examining the association between antibiotic use and depression [16–18, 29], but not including intermediate variables. These variables included maternal age (< 25, 25 to < 30, 30 to < 35, ≥ 35 years), pre-pregnancy body mass index (< 18.5, 18.5 to < 25, ≥ 25 kg/m^2^), highest education level (≤ 12, > 12 to ≤ 16, > 16 years), working status (employed or unemployed), annual household income (≤ 4, 4 to < 6, ≥ 6 million JPY), smoking status (never, quit before pregnancy, quit after pregnancy, current), alcohol intake (never, former, current) [30], parity (0, 1, ≥ 2), marital status (married or unmarried), psychiatric history of depression, anxiety disorder, dysautonomia, or schizophrenia (yes or no), and which of the 15 regional centers the participants belonged to. A directed acyclic graph (DAG) for this study is shown in Supplementary Fig. 1. Most variables were measured during early- to mid-pregnancy (i.e., median [IQR]: 15.4 [12.4, 19.4] weeks of gestation), and only two socioeconomic variables (i.e., educational background and income) were measured during mid- to late pregnancy (median [IQR]: 27.7 [25.4, 30.4] weeks of gestation). All variables were categorized according to standard clinical practice, common practice in Japan, and/or previous studies [31, 32].

### Statistical analysis

The mothers’ characteristics are summarized as frequencies and percentages.

A Bayesian multinomial generalized linear regression model, with a logit set as a link function and 15 regional centers as a random effect, was used to calculate crude and adjusted odds ratios (cORs and aORs) and their corresponding 95% credible intervals (CrIs) for the ordinal levels of psychological distress (0 = none; 1 = moderate distress; 2 = severe distress). Bayesian methods were adopted because the analysis required a multinomial model with random effects, and no suitable frequentist implementation in R was available. The “brms” package provided the necessary flexibility to fit this complex hierarchical structure. All of the potential confounders selected a priori were used to adjust the model with the forced entry method. In contrast, potential confounders were not used in the crude model. The group with no antibiotic use was set as the reference. A linear trend in antibiotic use was evaluated by treating antibiotic use patterns as a continuous variable—defined by the number of periods of use: 0 (no use), 1 (use during either period), or 2 (use during both periods)—using a Bayesian multinomial generalized linear regression model.

Multiple imputation was used to handle missing data. The missing value rate was ≤ 2% for all variables except for parity (2.54%), highest education level (3.50%), working status (4.26%), and annual household income (9.93%). Using chained equations [33], we created 20 imputed datasets by simultaneously entering all variables regardless of the missing value rates, and the results were integrated using Bayesian posterior distribution estimates.

To evaluate the robustness of the results, we conducted a complete case analysis as a sensitivity analysis (n = 78,324).

Data were analyzed using R (ver. 4.5.0; R Foundation for Statistical Computing, Vienna, Austria). Multiple imputation was performed using the “futuremice” function of the “miceadds” package (ver. 3.17–44). Bayesian multinomial logistic regression analysis and pooling of the results were performed using the “brm_multiple” function of the “brms” package (ver. 2.22.0).

## Results

A total of 94,490 mothers were analyzed. The mean age ± standard deviation (SD) was 30.8 ± 5.1 years, the mean pre-pregnancy BMI ± SD was 21.2 ± 3.3, and 41.5% of mothers were primiparous. Table 1 shows the participant characteristics. Compared with mothers who were included in the analysis (n = 94,490), those who were excluded (n = 2,902) tended to be older, to be non-smokers, to have higher levels of education and income, and to experience higher rates of miscarriage and stillbirth. The effective response rate was 97.0% (94,490/97,392).

**Table 1:** Characteristics of participants according to periconceptional antibiotic use pattern

		Antibiotic use					
		None		Either before or after pregnancy recognition		Both before and after pregnancy recognition	
		(*n* = 78,371)		(*n* = 14,853)		(*n* = 1,266)	
		n	(%)	n	(%)	n	(%)
Mother’s age at enrollment, y							
< 25		9,160	(11.7)	1,418	(9.5)	126	(10.0)
25 – < 30		23,010	(29.4)	4,143	(27.9)	379	(30.0)
30 – < 35		27,085	(34.6)	5,402	(36.4)	432	(34.2)
≥ 35		19,116	(24.4)	3,889	(26.2)	328	(25.9)
Pre-pregnancy body mass index, kg/m^2^							
< 18.5		12,704	(16.2)	2,419	(16.3)	234	(18.5)
18.5 – < 25		57,343	(73.2)	10,885	(73.3)	902	(71.2)
≥ 25		8,325	(10.6)	1,549	(10.4)	130	(10.3)
Parity							
0		33,789	(43.1)	6,215	(41.8)	460	(36.3)
1		28,911	(36.9)	5,803	(39.1)	554	(43.8)
≥ 2		15,671	(20.0)	2,835	(19.1)	253	(20.0)
History of major psychiatric disease							
No		72,346	(92.3)	13,589	(91.5)	1,105	(87.3)
Yes		6,025	(7.7)	1,264	(8.5)	161	(12.7)
Highest education level, y							
≤ 12		29,060	(37.1)	4,779	(32.2)	409	(32.3)
> 12– ≤ 16		32,633	(41.6)	6,518	(43.9)	572	(45.2)
> 16		16,679	(21.3)	3,557	(23.9)	285	(22.5)
Employment status							
No		29,387	(37.5)	5,354	(36.0)	403	(31.8)
Yes		48,984	(62.5)	9,499	(64.0)	864	(68.2)
Annual household income, JPY							
< 4 million		32,560	(41.5)	5,547	(37.3)	447	(35.3)
4– < 6 million		25,612	(32.7)	4,963	(33.4)	437	(34.5)
≥ 6 million		20,198	(25.8)	4,343	(29.2)	382	(30.2)
Smoking status							
Never		45,592	(58.2)	8,609	(58.0)	751	(59.3)
Quit before pregnancy		18,119	(23.1)	3,662	(24.7)	300	(23.7)
Quit after pregnancy		10,810	(13.8)	1,915	(12.9)	150	(11.9)
Current		3,850	(4.9)	667	(4.5)	65	(5.1)
Alcohol intake							
Never		27,223	(34.7)	4,954	(33.4)	378	(29.9)
Former		43,347	(55.3)	8,335	(56.1)	750	(59.2)
Current		7,802	(10.0)	1,564	(10.5)	138	(10.9)
Marital status							
Married		74,618	(95.2)	14,257	(96.0)	1,215	(96.0)
Unmarried		3,753	(4.8)	596	(4.0)	51	(4.0)

Values show the imputed data for the 94,490 expectant mothers

Table 2 shows prevalences and both crude and adjusted odds ratios (95% CrIs) for cases of moderate and severe psychological distress according to patterns of antibiotic use. Using no antibiotic use as a reference, the aORs (95% CrIs) for moderate psychological distress were 1.12 (1.07–1.16) for use during either the period before or after pregnancy recognition and 1.22 (1.08–1.38) for use during both periods. For severe psychological distress, the corresponding aORs (95% CrIs) were 1.07 (0.97–1.19) and 1.50 (1.15–1.94), respectively. A test for trend, based on the number of periods of antibiotic use, indicated a dose–response-like pattern for both moderate and severe psychological distress.

**Table 2:** Cases, prevalences, and crude and adjusted odds ratios (95% credible intervals) for two levels of psychological distress according to periconceptional antibiotic use pattern

	Antibiotic use			
	None	Either before or after pregnancy recognition	Both before and after pregnancy recognition	*p*-values for trend
	(*n* = 78,371)	(*n* = 14,853)	(*n* = 1,266)	
Non-cases, n	53,283	9,841	792	
Moderate psychological distress				
Cases, n	22,287	4,485	408	
Prevalence, %	29.5	31.3	34.0	
Crude odds ratio	1.00 (Ref.)	**1.09 (1.05, 1.13)**	**1.23 (1.09, 1.39)**	** < 0.001**
Adjusted odds ratio^a^	1.00 (Ref.)	**1.12 (1.07, 1.16)**	**1.22 (1.08, 1.38)**	** < 0.001**
Severe psychological distress				
Cases, n	2,802	527	66	
Prevalence, %	5.0	5.1	7.6	
Crude odds ratio	1.00 (Ref.)	1.02 (0.92, 1.12)	**1.56 (1.20, 2.01)**	**0.039**
Adjusted odds ratio^a^	1.00 (Ref.)	1.07 (0.97, 1.19)	**1.50 (1.15, 1.94)**	**0.006**

Moderate psychological distress: a K6 score of 5–12

Severe psychological distress: a K6 score of ≥ 13

Boldface type indicates that the 95% credible interval did not cross the reference (= 1.00)

^a^Adjusted for maternal age, pre-pregnancy body mass index, highest education level, working status, annual household income, smoking status, alcohol intake, parity, marital status, psychiatric history of depression, anxiety disorder, dysautonomia, and schizophrenia, with the 15 regional centers set as a random effect

No meaningful differences were observed in the results derived from the complete case analysis (Supplementary Table 1).

## Discussion

The purpose of this study was to clarify the relationship between mental health during pregnancy and the use of antibiotics, with a particular focus on extending the scope of antibiotic use to the preconception period from a preventive perspective regarding postpartum depression. To achieve this, we utilized the JECS dataset and analyzed 94,490 mothers, examining the association between patterns of antibiotic use (none, use during either period, or use during both periods) across two defined periods within the preceding 1-year interval––before and after pregnancy recognition, respectively––and the prevalence of moderate and severe psychological distress (K6) in early- to mid-pregnancy. The analysis was adjusted for potential confounding factors using a Bayesian modeling approach, which allowed for flexible modeling. The results revealed a general increase in psychological distress in early- to mid-pregnancy with a greater number of periods of antibiotic use (i.e., no use, use during either period, or use during both periods), irrespective of the severity of psychological distress. Thus, based on this dose–response-like pattern, periconceptional antibiotic use, even during a single period, emerged as an independent risk factor for psychological distress during early- to mid-pregnancy. This finding highlights the importance of appropriate antibiotic use starting from the preconception period for the prevention of postpartum depression.

The findings of this study, indicating an association between antibiotic use and increased psychological distress, are broadly consistent with previous antibiotic studies targeting pregnant women and postpartum mothers [17, 18]. Additionally, the results align well with two large-scale retrospective studies targeting the general population, each involving over 1 million participants [16, 34], and a prospective large-scale study involving over 300,000 participants [35]. A recent systematic review [15] found that all eight of the reviewed studies reported a relationship in the same direction. However, a recent retrospective cohort study [29], which was not included in the review, suggested an inverse association, pointing out that most of the evidence regarding antibiotics is derived from observational studies. This is because the issue of antibiotic-resistant bacteria makes it difficult to conduct randomized controlled trials on healthy individuals. In this regard, the present study is also an observational study and falls within this category. However, it has one distinctive feature: unlike previous studies, it focuses on pregnant women and examines antibiotic use in the year prior to pregnancy recognition. Additionally, although this study shares a common focus with the previous study [18] in terms of using maternal mental health during pregnancy as an outcome, it employs an adjustment model specific to antibiotic use, thereby clarifying the relationship with reduced bias. Therefore, we believe that our findings contribute to the existing body of knowledge.

The mechanism linking antibiotic use to psychological distress likely involves a pathway mediated by the gut microbiota. Antibiotic use disrupts the gut microbiota in various ways [20, 36], and such disruptions can influence brain function through bidirectional pathways involving the autonomic nervous system, neuroendocrine system, gut–brain axis, and immune system [21]. These include gut barrier dysfunction, which can lead to cytokine release and subsequently to inflammation-mediated worsening of depression [37]; reduced production of depression-related neurotransmitters and neuropeptides, such as gamma-aminobutyric acid (GABA), serotonin, and brain-derived neurotrophic factor (BDNF) due to decreased levels of lactobacilli and bifidobacteria [36]; and a decrease in lactobacilli, which modulates γδ T cells involved in intestinal immunity, ultimately contributing to increased depression [38]. In fact, patients with major depressive disorder have lower levels of lactobacilli and bifidobacteria compared with healthy controls [39], and disparities in the α-diversity and β-diversity of the gut microbiota have been observed [40]. A recent study revealed that women with children aged 0 to 4 years who have higher α-diversity of the gut microbiota show fewer depressive symptoms [41]. Therefore, although we did not directly examine the gut microbiota in this study, such changes might have occurred in antibiotic users. Interestingly, in contrast to the findings of the present study, meta-analyses have reported that probiotics may improve depression [42]. During pregnancy, changes specific to pregnant women, including changes in food preferences, may also occur. Therefore, future research considering these factors is warranted.

As previously mentioned, in addition to the results of this study, there is epidemiological evidence linking antibiotics to psychiatric disorders [15]. During pregnancy, women are at increased risk of various infections, including urinary tract infections [43]. Combined with the increased likelihood of detecting infections such as genital chlamydia, syphilis, toxoplasmosis, gonorrhea, bacterial vaginosis, and group B streptococcus during routine pregnancy checkups [44], the reliance on antibiotics has tended to increase. In fact, a Canadian study involving over 200,000 pregnant women found that antibiotic prescriptions for pregnant women accounted for the top 1 or 2 most commonly used medications during pregnancy, and the frequency of use was reported to be very high immediately postpartum [14]. Given the need for caution in using antidepressants during pregnancy [45, 46], and the vulnerability of women’s mental health during this period [13, 47], optimizing antibiotic use from preconception to the early pregnancy period is important not only for addressing antibiotic resistance but also for conducting preconception care that reduces or prevents perinatal depression [8].

This study has several strengths. First, it includes mothers from 15 regional centers across Japan, which enhances the generalizability of the findings to a broader Japanese population. Second, with over 94,000 mothers included, the large sample size enables the calculation of estimates with high precision. Third, the exclusion rate due to missing data on antibiotic use was low at 3%, allowing most cases to be included in the analysis, thereby reducing the possibility of selection bias.

This study also has several limitations. First, it did not specify the types of antibiotics used. Although antibiotics have different spectra, this study did not distinguish among them. Second, this study did not specify the reasons for use or the route of administration. Oral administration is commonly used for mild to moderate infections, whereas intravenous or injection administration is often used for severe infections or hospitalized patients; however, this study did not distinguish between these forms. Third, the time interval investigated for antibiotic use and the point at which psychological distress was assessed were closer than in previous studies [16]. Consequently, for some participants, there may not have been sufficient time for psychological distress to manifest, which could lead to underestimation of the associated risk. Fourth, to investigate dose–response-like patterns by targeting multiple time periods, as in previous studies, the number of periods of antibiotic use (0, 1, and 2) was relatively limited compared with a previous study that used 4 levels: 0, 1, 2–5, and ≥ 5 courses [16]. Similarly, the amount of use was not assessed. Therefore, although a rough dose–response-like pattern was observed, a more specific association could not be verified. Fifth, although antibiotic use was assessed using an interviewer-administered questionnaire, mothers were asked to recall all medications (not limited to antibiotics) they had used, which might have introduced recall bias and led to an underestimation of antibiotic use. Sixth, while validated questionnaires were used to measure psychological distress, these do not correspond to clinical diagnoses of depression or anxiety. As a result, cases might have been overestimated compared with those obtained using clinical diagnostic criteria [48], and other cases might have been missed. Seventh, despite incorporating various potential confounders into the model, we cannot exclude the existence of unmeasured potential confounders, such as the effect of the infection itself that led to antibiotic use. Consequently, we may be overestimating the associated risk. Finally, we adjusted the model using a history of depression (as a psychiatric disorder) to prevent potential reverse causality. However, this adjustment did not necessarily rule out the occurrence of reverse causality. Because patients with depression tend to receive more antibiotic prescriptions [49], future studies will require variable acquisition to better guard against the possibility of reverse causality.

In conclusion, this study demonstrated that antibiotic use during the past year prior to early pregnancy—even if limited to a single period—was independently associated with psychological distress during early- to mid-pregnancy, following a dose–response-like pattern. This finding highlights the importance of judicious antibiotic use beginning in the preconception period in order to help reduce or prevent postpartum depression.

## Supplementary Information

Supplementary Material 1.

## Data Availability

Data are unsuitable for public deposition due to ethical restrictions and the legal framework of Japan. It is prohibited by the Act on the Protection of Personal Information (Act No. 57 of 30 May 2003, amendment 9 September 2015) to publicly deposit data containing personal information. Ethical Guidelines for Medical and Health Research Involving Human Subjects enforced by the Japanese Ministry of Education, Culture, Sports, Science and Technology and the Ministry of Health, Labour and Welfare also restrict the open sharing of epidemiologic data. All inquiries about access to data should be sent to jecs-en@nies.go.jp. The person responsible for handling inquiries sent to this e-mail address is Dr Shoji F. Nakayama, JECS Programme Office, National Institute for Environmental Studies.

## Abbreviations

JECS: Japan Environment and Children’s Study
BMI: Body mass index
K6: Kessler Psychological Distress Scale
aOR: Adjusted odds ratio
cOR: Crude odds ratio
CrI: Credible interval
