# Supplementary material for: Periconceptional antibiotic use and early- to mid-pregnancy psychological distress in a nationwide birth cohort: cross-sectional analysis from the Japan Environment and Children’s Study
Source: BMC Public Health. 2026 Jan 10;26:863. doi: 10.1186/s12889-025-26119-0 (PMC12977711; doi:10.1186/s12889-025-26119-0)
Supplement: Supplementary file 1 — Supplementary Material 1. [file 12889_2025_26119_MOESM1_ESM.docx]

**Supplementary Material**

**Periconceptional antibiotic use and early pregnancy psychological distress in a nationwide birth cohort: Cross-sectional analysis from the Japan Environment and Children’s Study**

Kenta Matsumura^1*,2,3^; Hitomi Inano^3,4^, Junko Sakai^3^, Kanako Shimada^3^, Akiko Tsuchida^2,3^; Hidekuni Inadera^2,3^; the Japan Environment and Children’s Study (JECS) Group^5^

**Table of Contents**

**Supplementary Figure 1**

**Supplementary Table 1**

**Supplementary Figure 1.** A directed acyclic graph (DAG).

**Supplementary Table 1.** Cases, prevalences, and crude and adjusted odds ratios (95% credible intervals) for two levels of psychological distress according to periconceptional antibiotic use pattern (complete case analysis).

|  |  | Antibiotic use |  |  |  |
| --- | --- | --- | --- | --- | --- |
|  |  | None | Either before or after pregnancy recognition | Both before and after pregnancy recognition | *p*-values for trend |
|  |  | (n = 64,712) | (n = 12,542) | (n = 1,070) |  |
| Non-cases, n | | 44,402 | 8,347 | 675 |  |
| Moderate psychological distress | | |  |  |  |
|  | Cases, n | 18,143 | 3,774 | 339 |  |
|  | Prevalence, % | 29.0 | 31.1 | 33.4 |  |
|  | Crude odds ratio | 1.00 (Ref.) | **1.11 (1.06, 1.15)** | **1.23 (1.08, 1.40)** | **<0.001** |
|  | Adjusted odds ratio^a^ | 1.00 (Ref.) | **1.13 (1.09, 1.18)** | **1.21 (1.06, 1.38)** | **<0.001** |
| Severe psychological distress | | |  |  |  |
|  | Cases, n | 2,167 | 421 | 56 |  |
|  | Prevalence, % | 4.7 | 4.8 | 7.7 |  |
|  | Crude odds ratio | 1.00 (Ref.) | 1.03 (0.93, 1.15) | **1.69 (1.28, 2.20)** | **0.018** |
|  | Adjusted odds ratio^a^ | 1.00 (Ref.) | 1.09 (0.97, 1.21) | **1.61 (1.19, 2.14)** | **0.002** |

Moderate psychological distress: a K6 score of 5–12.

Severe psychological distress: a K6 score of ≥13.

**Boldface type** indicates that the 95% credible interval did not cross the reference (=1.00).

^a^ Adjusted for maternal age, pre-pregnancy body mass index, highest education level, working status, annual household income, smoking status, alcohol intake, parity, marital status, psychiatric history of depression, anxiety disorder, dysautonomia, and schizophrenia, with the 15 regional centers set as a random effect.
